# Design, Analysis, and Verification of Ka-Band Pattern Reconfigurable Patch Antenna Using RF MEMS Switches

**DOI:** 10.3390/mi7080144

**Published:** 2016-08-17

**Authors:** Zhongliang Deng, Xubing Guo, Hao Wei, Jun Gan, Yucheng Wang

**Affiliations:** School of Electronic Engineering, Beijing University of Posts and Telecommunications, Beijing 100876, China; dengzhl@bupt.edu.cn (Z.D.); haowei@bupt.edu.cn (H.W.); GanJ@bupt.edu.cn (J.G.); wangyucheng@bupt.edu.cn (Y.W.)

**Keywords:** pattern reconfigurable antenna, RF MEMS switch, far-field addition model, Ka-band

## Abstract

This paper proposes a radiating pattern reconfigurable antenna by employing RF Micro-electromechanical Systems (RF MEMS) switches. The antenna has a low profile and small size of 4 mm × 5 mm × 0.4 mm, and mainly consists of one main patch, two assistant patches, and two RF MEMS switches. By changing the RF MEMS switches operating modes, the proposed antenna can switch among three radiating patterns (with main lobe directions of approximately −17.0°, 0° and +17.0°) at 35 GHz. The far-field vector addition model is applied to analyse the pattern. Comparing the measured results with analytical and simulated results, good agreements are obtained.

## 1. Introduction

Radiating pattern reconfigurable antennas for the design of high-capacity systems have obtained increasing popularity, owing to their attractive advantages, such as compact structure and high efficiency. Pattern reconfigurable antenna could change the maximum radiating direction while maintaining unchanged operating frequencies and other performances. To date, many reconfigurable antennas have been proposed by using PIN diodes [1], copper strip [2], and other means. However, compared with other reconfigurable technologies, RF micro-electromechanical systems (RF MEMS) technology has many superiorities over others, such as small size, high quality factors, and high linearity [3]. RF MEMS switches have been used broadly in reconfigurable antennas in recent years [4,5,6,7,8,9], but most of those antennas work in low frequencies and have no process consistence (i.e., RF MEMS switches were mounted in circuits after the antenna was manufactured). When the operating frequency rises, the dielectric loss increases, and the transmission coefficient is more sensitive to the variations of the dielectric thickness and dielectric constant. Moreover, the size of RF MEMS switches and operating wavelengths have the same order of magnitude, and the RF MEMS switches will introduce some parasitic capacitances. The challenges of designing high frequency RF MEMS antennas as compared to low frequency antennas are the radiation efficiency, the transmission characteristics, and the robustness of overall antenna. There are many advantages of Ka-band over lower frequency, such as wide bandwidth, less interference, compact device structure, etc. Compared with lower frequency antennas (such as C-band or X-band antennas), Ka-band antennas have powerful data throughput capacity and the advantage of compact structure. These antennas can be applied to the 5th-generation (5G) mobile communication and satellite communication system.

We have done some research in reconfigurable antennas [10,11] and have fabricated several kinds of reconfigurable antennas in the same batch. Those antennas all have different structure configuration and size, and two of them can be found in references [10,11]. However, [10] only presents the structure configuration and measurement results, and [11] is only a simulation. Little work has been done on the principles of the proposed reconfigurable antenna and the design details of the proposed RF MEMS switch, such as the actuating voltage, pull in time, and fabrication process. This paper gives details on the design, analysis, and verification of a Ka-band pattern reconfigurable patch antenna. In addition, the principles of the proposed reconfigurable antenna, the research of relationship between resonant frequency and RF MEMS switch structure configuration, the actuating voltage, the pull in time, and the fabrication process of the proposed RF MEMS switch are included.

In this paper, a pattern reconfigurable antenna operating at 35 GHz is presented. By changing the mode of the RF MEMS switches, the antenna is capable of switching among four operating modes and obtaining three operating states (because two of them are at the same angle, i.e., 0°). By changing the connections between the main and assistant patch radiator, the beams can be steered in three different directions in the *yoz* plane (ϕ = 90°).

The article is organized as follows. Section 2 illustrates the designs of the antenna and RF MEMS switches; Section 3 describes the theory analysis and operation mechanism of the pattern property of the proposed antenna; Section 4 provides the results and discussion; Section 5 summarizes and concludes this study.

## 2. Pattern Reconfigurable Antenna Structure and Design

### 2.1. Overall Structure and Design

**Figure 1:**
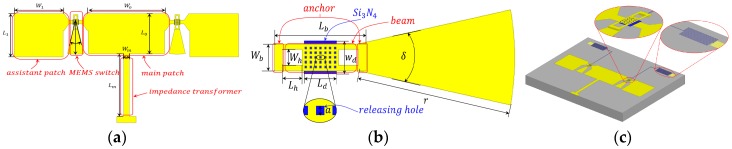
Proposed radiating pattern reconfigurable antenna using micro-electromechanical systems (MEMS) switches. (**a**) Structure of the reconfigurable antenna; (**b**) Structure of the switch used in the reconfigurable antenna; (**c**) Overall structure of the proposed antenna.

A strategy for a radiating pattern reconfigurable antenna is proposed, the structure is illustrated in Figure 1a, and the optimized geometry of the RF MEMS switch is shown in Figure 1b. The pattern reconfigurable antenna—fabricated on a high resistivity silicon substrate with a relative dielectric constant of 11.9 and thickness of 400 μm—consists of one rectangular main patch radiator, two rectangular assistant patch radiators, one impedance transformer, and two RF MEMS switches. The RF MEMS switch is made of an anchor, a beam, and a *λ_g_*/4 sector open stub. All the conductor patches and RF MEMS beams are made of aluminum. The optimized configurations of the antenna and switches are shown in Table 1.

In general, the microstrip antenna operates at a resonant state. Thus, the length of the radiator is selected to be approximately half the waveguide wavelength. The relationship between the length of the radiators and the resonant frequency can be summarized as follows [12].

(1) $$\left\{ \begin{array}{l} L=\frac{1}{2}\frac{c}{f_{res}\sqrt{\varepsilon_{eff}}}\\ \varepsilon_{eff}=\frac{\varepsilon_{r}+1}{2}+\frac{\varepsilon_{r}-1}{2}{\left(1+\frac{10h}{W}\right)}^{-\frac{1}{2}} \end{array}\right.$$

where, *c* is the velocity of light in free space, *f_res_* is the resonant frequency, *h* is the substrate thickness, *W* is the width of microstrip patch, and *ε_r_* and *ε_eff_* are the relative and effective dielectric constants, respectively.

It is mentioned that the actual length of the radiator patch is optimized by simulation software and is not exactly equal to half of the waveguide wavelength, for the sake of gaining the best performance of overall structure. By controlling the states of different RF MEMS switches, the proposed antenna can switch among four operating states, and obtain three reconfigurable radiating patterns, as shown in Table 2.

According to the above measurement results of the main lobe direction, the antenna can mainly achieve three reconfigurable radiating patterns—i.e., left (approximately −17°), middle (approximately 0°), and right (approximately +17°), respectively.

### 2.2. RF MEMS Switch Structure and Design

**Figure 2:**
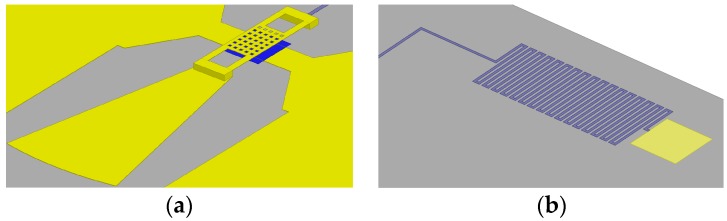
RF MEMS switch. (**a**) 3D model. (**b**) Bias line and pad.

The RF MEMS switch plays a vital role in the pattern reconfigurable antenna. RF MEMS switches have very low loss, they do not consume any direct current (DC) power, and their linearity is excellent [13,14]. The optimized geometry is shown in Figure 1b and Figure 2a.The top view of the proposed RF MEMS switch is shown in Figure 1b, and the size is shown in Table 1. The beam thickness is 1 μm. The air gap between the RF MEMS switch beam and the signal lines is 1.5 μm. By varying this air gap, the switch can work in the up or the down state and determine whether the assistant patch is connected to the main patch (i.e., when the switch is in the down state, the assistant patch is detached from main patch, and vice versa).

The RF MEMS switches were terminated by a *λ_g_*/4 sector open stub and a high resistivity bias line, shown in Figure 2a. The *λ_g_*/4 sector open stub is used to transform the open circuit to a short circuit for fear of causing a great impact on the resonant frequency and return loss of the antenna [15]. The advantages of a sector open stub over a microstrip line are miniaturization of structure and convenience of connection. The bias line is applied by direct current voltage to actuate the RF MEMS switch, as shown in Figure 2b.

**Figure 3:**
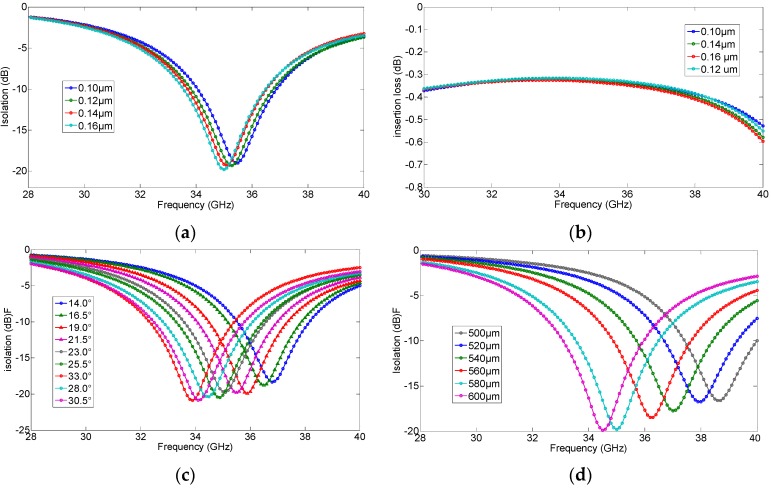
The relationship between resonant frequency (when the switch in the down state) and structure parameters. (**a**) The variation of the thickness of dielectric (isolation); (**b**) The variation of the thickness of dielectric (insertion loss); (**c**) The variation of the angle of the sector open stub; (**d**) The variation of the radius of the sector open stub.

The dielectric shown in Figure 2a is Si_3_N_4_ with the thickness 0.16 μm. The thickness of dielectric, the degree and radius of sector open stub are all related to the resonant frequency of the RF MEMS. In order to select the optimized parameters of the RF MEMS switch, some simulations of the switch were conducted under the conditions of various parameter combinations. The resonant frequency of the RF switch (in the down state) raises with the increase of the degree of sector stub, with the increase of the dielectric thickness and with the decrease of the radius of the sector stub, as shown in Figure 3. According to the requirement of optimal isolation and the antenna performance operating at 35 GHz, the combination of parameters is determined (i.e., the angle of the sector open stub is 25°, the dielectric thickness is 0.16 μm, and the radius of sector open stub is 580 μm).

The isolation of the RF MEMS switch in down state reaches 20 dB at an operating frequency of 35 GHz, and it can guarantee the performance of reconfigurable antenna. When the switch is in the up state, the resonant frequency of the switch is very high (beyond 40 GHz) and has slight changes with variations of the parameters, as shown in Figure 3b. Thus, only the relationship between structure configuration parameters of the up state switch and resonant frequency is analyzed without loss of generality.

When the position of the switch beam reaches the point $\frac{2g_{0}}{3}$, the increase of the electrostatic force is greater than the increase of the restoring force, resulting in a rapid drop-down of the beam. The actuating voltage corresponding this position is maximum. To fully pull the switch down, the actuating voltage must be equal to or greater than the maximum voltage. The actuating voltage of the switch can be evaluated by formula

(2) $$V_{p}=\sqrt{\frac{2k_{e}}{\varepsilon_{0}W_{b}L_{d}}\frac{g_{0}}{3}{\left(\frac{2g_{0}}{3}+\frac{t_{e}}{\varepsilon_{r}}\right)}^{2}}$$

where *k_e_* is the effective elastic coefficient, *ε*_0_ is the dielectric constant in free space, *g*_0_ is the air gap between the RF MEMS switch beam and the Si_3_N_4_ dielectric, *ε_r_* is the relative dielectric constant of Si_3_N_4_ dielectric, *t_e_* is the thickness of the RF MEMS switch beam, *W_b_* is the width of the MEMS switch beam, and *L_d_* is the length of the Si_3_N_4_ dielectric, respectively. The actuating voltage (approximately 7.5 V) is evaluated by using Formula (2). However, the measured actuating voltage is 15.8 V—approximately twice the calculation value using Formula (2). This is mainly caused by the incomplete release of polyimide and the inhomogeneity of thickness. When the manufacture process has a good release and flatness, the actuating voltage will decrease to the theoretical value. The quality factor [15] of the RF MEMS switch is *Q =* [4*ρt_e_*^2^*E*^−1/2^/*μ*(*W_b_L_d_*)^2^]*g*_0_^3^ ≈ 1. Thus, the pull in time of the RF MEMS switch is *t_s_ ≈* (*27V_p_*^2^)/(*4ω*_0_*QV_s_*^2^) ≈ 12.6 μs, where *E* is Young’s modulus, *μ* is the air viscosity coefficient between the MEMS beam and the Si_3_N_4_ dielectric, and *ω*_0_ is the mechanical resonant frequency, respectively.

The overall structure of the antenna and the RF MEMS switches is fabricated on a 400 μm-thick high resistivity silicon substrate, and then 0.3 μm thickness of SiO_2_ is formed by thermal oxidation as an insulating layer. To form the Coplanar Waveguide (CPW) transmission lines, 0.2 μm thickness of Al is deposited and patterned to define DC bias pads afterward. Thin SiAl (~0.05 μm) is patterned by lift off to form the bias lines after deposition. Plasma Enhanced Chemical Vapor Deposition (PECVD) 1500 Å thickness of Si_3_N_4_ is patterned on top of the bottom electrode and bias lines. One point five micron thickness of Al is evaporated as the anchors. Polyimide (as the sacrificial layer) was cut down from 5 μm to less than 1.3 μm by chemical mechanical polishing (CMP). The beam uses 0.6 μm of SiAl. Finally, the wafer is released in a plasma dryer to avoid collapsing the membrane.

## 3. Theory Analysis

**Figure 4:**
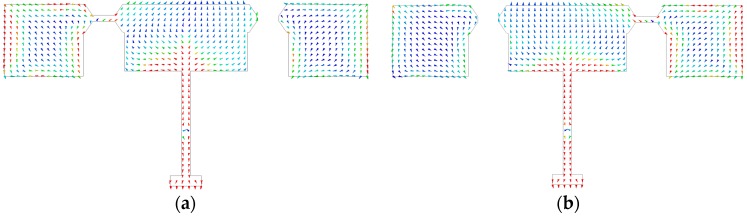
Distribution of surface current density of antenna. (**a**) Left switch in the up state and right in the down state; (**b**) Left switch in the down state and right in the up state. (Note that switches were replaced by conductor patch without loss of generality)

A prevalent method to analyze the directivity of antennas is to investigate the surface electric currents ***J****_s_* on the patch, and then one can calculate the radiating pattern using the weighted function *U*(*θ*, ϕ) of antenna directivity [16]. The plots of surface currents density are shown in Figure 4 for two different states (the relative value of surface currents represented by rainbow color, red and violent explicate maximum and minimum, respectively). However, understanding the radiation from these currents requires extra effort, since the surface currents density distribution ***J_s_*** is unknown and is hard to obtain. Furthermore, the integral in the region where ***J_s_*** exists is hard to solve

(3) $$\left\{ \begin{array}{l} U\left(\theta ,\varphi \right)=\int J_{s}\left(x^{\prime },y^{\prime },z^{\prime }\right)e^{j\Omega }dv^{\prime }\\ \Omega =k_{0}\left(x^{\prime }\sin\theta \cos\varphi +y^{\prime }\sin\theta \sin\varphi +z^{\prime }\cos\theta \right) \end{array}\right.$$

where, *U*(*θ*, ϕ) is the weighted function of antenna directivity, *k_0_* is the wavenumber in free space, (*x′*, *y′*, *z′*) is the coordinate of the source point, *v′* represents the region where ***J_s_*** exists, and (*θ*, ϕ) is the angle in the spherical coordinate frame, respectively.

**Figure 5:**
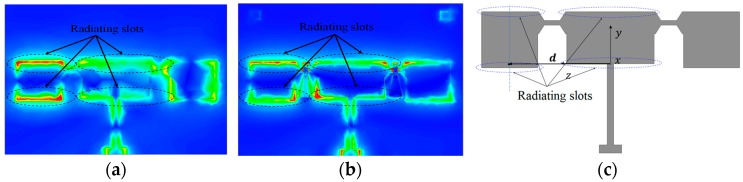
Distribution of the surface electric field of the antenna, when the left switch is in the up state and the right is in the down state. (**a**) Switches were replaced by a conductor patch; (**b**) Using RF MEMS switches; (**c**) The coordinate frame for field calculation. The relative value of surface electric field was represented by rainbow color, red and violent represent maximum and minimum respectively.

Instead, plotting the electric fields on the patch, the electric field vector addition method is employed in the far-field region to evaluate far-field distribution. Figure 5a shows the distribution of the electric field while the RF MEMS switches were replaced by conductor patches, in order to simplify the complexity of radiating problems. In contrast, the electric field distribution of an antenna using RF MEMS switches was demonstrated in Figure 5b, and one can conclude that the electric field distribution is almost unchanged by RF MEMS switches. The differences between Figure 5a and Figure 5b are mainly caused by the impact of sector stubs, but the maximum of the distribution of the electric field is almost unchanged and has no effect on the analysis process for the rest of this paper. Hence, the far-field of the antenna will be estimated by using conductor-connected patches instead of RF MEMS switches. It is easy to conclude that the far-field radiation is principally produced by upper and lower slots of main and assistant radiating patches (shown in Figure 5), when the left switch is in the up state and right is in the down state. The same conclusion can be applied to other modes where the switches were in the different states.

To simplify, the radiating field was evaluated by vector field addition of the upper and lower slots of the main and assistant radiating patches. The antenna radiation dominated by radiating slots is annotated in Figure 5a,b, and the far-field can be calculated by equivalent magnetic current model [17]. The electrical vector potential generated by equivalent magnetic currents of main and assistant patch radiating slots are ***F_m_*** and ***F_a_*_,_** respectively

(4) $$\left\{ \begin{array}{l} F_{m}\propto -\hat{z}\frac{1}{R}e^{-jk_{0}R}\cdot \frac{\sin\left(k_{0}h\sin\theta \cdot \cos\varphi \right)}{k_{0}h\sin\theta \cdot \cos\varphi }\cdot \frac{\sin\left(\frac{1}{2}k_{0}w_{m}\cos\theta \right)}{k_{0}\cos\theta }\\ F_{a}\propto -\hat{z}\frac{1}{\left|R-d\right|}e^{-jk_{0}\left|R-d\right|}\cdot \frac{\sin\left(k_{0}h\sin\theta \cdot \cos\varphi \right)}{k_{0}h\sin\theta \cdot \cos\varphi }\cdot \frac{\sin\left(\frac{1}{2}k_{0}w_{a}\cos\theta \right)}{k_{0}\cos\theta } \end{array}\right.$$

where (*R*, *θ*, ϕ) is the observation point coordinate in the spherical coordinate frame, *k*_0_ is the wavenumber in free space, *h* is the height of the substrate, ***R*** is the vector from origin of the spherical coordinate frame to the observation point, ***d*** is the vector annotated in the Figure 5c, and *w_m_* and *w_a_* represent the width of main patch (*W*_0_) and the assistant patch (*W*_1_) as shown in Figure 1a, respectively.

Correspondingly, the far-field distribution is determined by electromagnetic duality, and the electric field is

(5) $$E_{\varphi }=\left[-\nabla \times \left(F_{m}+F_{a}\right)\right]\propto \left\{\begin{array}{l} j\frac{1}{R}e^{-jk_{0}R}\cdot \frac{\sin\left(k_{0}h\sin\theta \cdot \cos\varphi \right)}{k_{0}h\sin\theta \cdot \cos\varphi }\cdot \frac{\sin\left(\frac{1}{2}k_{0}a\cos\theta \right)}{a\cos\theta }\sin\theta +\\ j\frac{1}{\left|R-d\right|}e^{-jk_{0}\left(\left|R-d\right|-\frac{1}{2}b\sin\theta \cdot \sin\varphi \right)}\cdot \frac{\sin\left(k_{0}h\sin\theta \cdot \cos\varphi \right)}{k_{0}h\sin\theta \cdot \cos\varphi }\cdot \frac{\sin\left(\frac{1}{2}k_{0}a\cos\theta \right)}{a\cos\theta }\sin\theta \cdot \cos\left(\frac{1}{2}k_{0}b\sin\theta \sin\varphi \right) \end{array}\right\}$$

**Figure 6:**
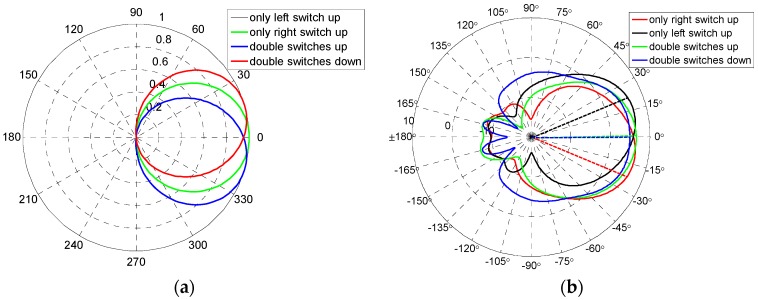
Radiation patterns of reconfigurable antenna. (**a**) Theoretical evaluation of patterns; (**b**) Simulation of patterns.

The magnetic field can also be expressed according to the electromagnetic relationship of plane wave in the free space or Maxwell equations. Hereto, the radiating patterns were obtained and can be plotted using MATLAB software (MATLAB 7.10, The Mathworks, Natick, MA, USA) as shown in Figure 6a. Comparing the theoretical evaluation patterns with the simulation patterns, good agreements of maximum radiating directions are obtained. However, pattern simulation results have back lobe because of coupling between the main and assistant patches. Due to ideal assumptions of the radiating model, the theoretical evaluation pattern has no back lobe. However, the back lobe of the patterns have no influences on maximum radiating direction of the reconfigurable antenna—i.e., the effects of the back lobe can be neglected when one focuses on the maximum radiating direction.

## 4. Results and Discussion

**Figure 7:**
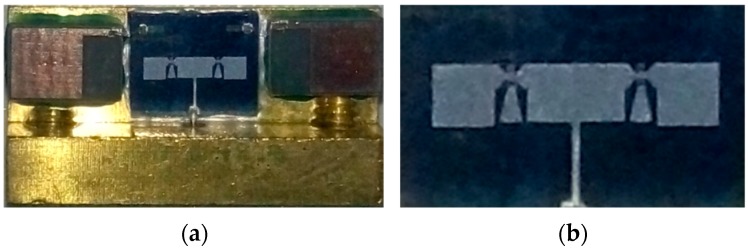
Photograph of the proposed antenna. (**a**) Antenna with testing holder; (**b**) Pure antenna fabricated on high resistivity silicon.

In this paper, the proposed pattern reconfigurable antenna was simulated by HFSS software (HFSS13.0, Anosoft, Pittsburgh, PA, USA), and the results are presented in Figure 6b. Photographs of the antenna are shown in Figure 7. The proposed antenna was measured by the network analyzer Agilent PNA N5442A (10 MHz–43.5 GHz) (Agilent, Santa Clara, CA, USA).

### 4.1. Input Impedance Matching of All Modes

**Figure 8:**
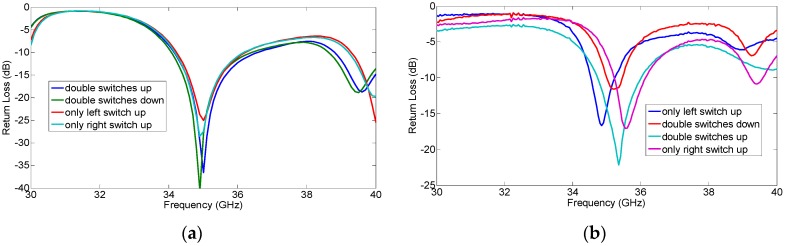
The measured return losses of all modes. (**a**) Simulation; (**b**) Measurement.

It is known that the input impedance matching of all modes at the desired frequency 35 GHz is essential to the resonant antenna. In this article, the antenna is fed by a 50 Ω microstrip line, and the input impedance is transformed by a microstrip impedance transformer. The simulated return loss of all modes at the desired frequency are all greater than 20 dB, as shown in Figure 8a. As shown in Figure 8b, the measured return losses of all modes are greater than 10 dB. The discrepancies between the measured results and the simulated ones are mainly caused by conductor loss and the manufacture process, such as the lithographic resolution, the residual polyimide, the inhomogeneity of thickness, and the asymmetry between the left RF MEMS switch and the right RF MEMS switch. However, the shapes of the curves in Figure 8a,b are similar, and the measured return loss reached 10dB at desired frequency. Thus, the manufactured antenna has acceptable return loss curves. The proposed antenna is in impedance matching and resonant states at all pattern reconfigurable modes.

### 4.2. Pattern Reconfigurable Characteristics

**Figure 9:**
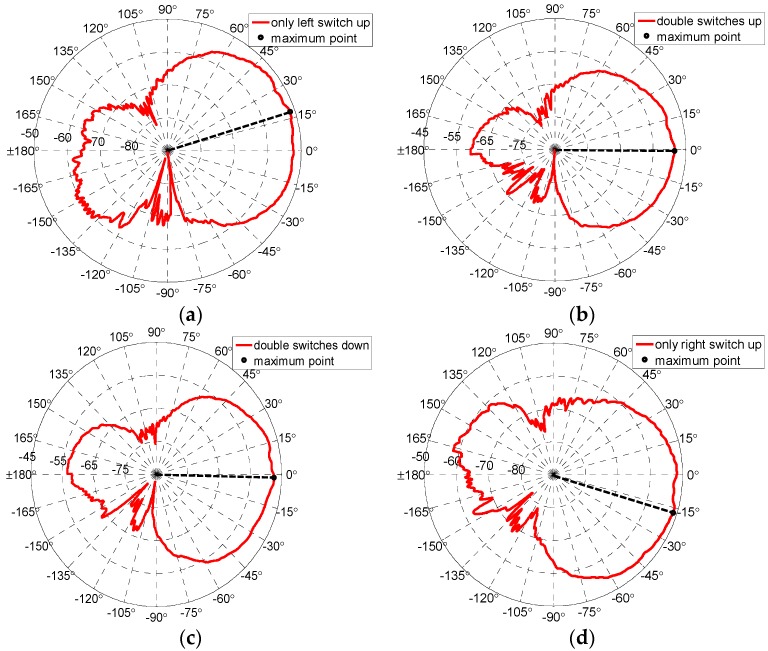
Measurement patterns of reconfigurable antenna for four modes. (**a**) Mode 1; (**b**) Mode 2; (**c**) Mode 3; (**d**) Mode 4.

Figure 9 gives the curves of measured radiating patterns of reconfigurable antenna. As shown in Figure 9, the proposed antenna can switch among four operating modes (−17.2°, −0.6°, −1.5°and 17.3°) and obtain three reconfigurable radiating patterns—i.e., left (approximately −17°), middle (approximately 0°), and right (approximately +17°), respectively.

The simulated patterns of the reconfigurable antenna are shown in Figure 6b. Comparing the simulated results with measured results, good agreements can be achieved. The errors in fabrication, such as the resolution of lithographic, the residual polyimide, thickness inhomogeneity, the asymmetry between the left RF MEMS switch and the right RF MEMS switch, and the conductor loss are the primary causes for any discrepancies observed in the measurements. When the manufacture process has a good release and flatness, the discrepancies will decrease rapidly. The measured patterns have back lobes due to coupling between the main and assistant patches.

### 4.3. Advancements

A performance comparison of the proposed pattern reconfigurable antenna with available literature is shown in Table 3. The compared results show that the proposed reconfigurable antenna has the advantages of more compact structure than the ones in available literature. In addition, the proposed reconfigurable antenna also has an acceptable range of reconfigurable angle, higher operating frequency, and the proposed pattern reconfigurable antenna does not need array architecture.

## 5. Conclusions

This paper introduces the design and analysis of a Ka-band pattern reconfigurable patch antenna using RF MEMS switches. A planar antenna with a pattern reconfigurable characteristic is presented for the sake of verification of proposed method. By changing the states of the RF MEMS switches, three reconfigurable radiating patterns are obtained—i.e., left (approximately −17°), middle (approximately 0°), and right (approximately +17°), respectively. Due to the small size and lightweight characteristics, the proposed pattern reconfigurable antenna is an excellent candidate for satellite searching, tracing, and communication systems.

## Figures and Tables

**Table 1:** Optimized configurations of the antenna and switches.

Symbol	Description	Value
*W*_0_	width of the main patch	1500 μm
*L*_0_	length of the main patch	900 μm
*W*_1_	width of the assistant patch	960 μm
*L*_1_	length of the assistant patch	970 μm
*W_m_*	width of the impedance transformer	110 μm
*L_m_*	length of the impedance transformer	1410 μm
*W_b_*	width of the switch beam	90 μm
*L_b_*	length of the switch beam	340 μm
*W_h_*	width of the hole	54 μm
*L_h_*	length of the hole	72 μm
*a*	side length of releasing hole	8 μm
*r*	length of the sector stub	575 μm
*δ*	angle of the sector stub	25°

**Table 2:** Four operating states and three reconfigurable radiating patterns.

Mode	State of RF MEMS Switches	Frequency	Main Lobe Direction (ϕ = 90°)
1	Only left switch in the up state	35 GHz	*θ* = 17.3°
2	Double switches in the up state	35 GHz	*θ* = −0.6°
3	Double switches in the down state	35 GHz	*θ* = −1.5°
4	Only right switch in the up state	35 GHz	*θ* = −17.2°

**Table 3:** Performance comparison of the proposed antenna with available literature.

Literature	Means	Arrays	Frequency	Reconfigurable Angles	Block Volume (mm^3^)
[18]	PIN diodes	Need	27.5 GHz	45°	5.1 × 5.1 × 1.274
[19]	MEMS switches	Need	34.8 GHz	60°	About 500 × 500 × 2
[20]	PIN diodes	No need	27 GHz	60°	9.5 × 9.5 × 1
[21]	MEMS switches	Need	30 GHz	13°	About 7.112 × 3.556 × 40
This paper	MEMS switches	No need	35 GHz	34°	2.45 × 4.4 × 0.4

PIN diodes: Positive intrinsic negative diodes.
